# Visual Factors in Equilibration, Especially Aviation

**Published:** 1918

**Authors:** 


***Visual Factors in Equilibration, Especially Aviation.*** By Percy FRIDENBERG. Abs. from *Journal. A. M. A.*, April 6, 1918.

The author believes that other factors than a normal labyrinth, which has generally been assumed to be the essential in equilibration, should be taken into consideration, and especially visual factors, which merit more attention in determining physical fitness than has so far been paid them. Clean vision and normal ocular muscle balance are especially necessary.

Forms of dizziness that may render a man unfit to control an aeroplane may result from neurasthenia, anemia, chronic alcoholism, malaria, chronic nicotine poisoning, and many other toxic states, as well as in chronic intestinal indigestion, lead intoxication, and convalescence from various diseases. Apparently healthy subjects complain also of dizziness due to various ocular anomalies, among which may be cited mixed astigmatism, accommodation spasm and paresis, ocular and motor imbalance, retinal hyperesthesis, and asthenopic strain from whatever cause, functional, organic, or, finally, exterior, such as defective or improper illumination, position, or work, faulty or ill-fitting glasses, especially prisms or cylinders, all of which defects are quite sufficient to cause dizziness to an incapacitating degree.

Labyrinthine disease is not common, and patients suffering from it are critically ill. The same may be said of dead labyrinths. No person suffering from such disorders is likely to be a candidate for flying.

Extreme or irregular stimulation of the organ of vision, dazzling or blinding, may cause dizziness to the point of complete lack of balance and loss of consciousness.

Irregular illumination and motion of objects alone are sufficient to cause dizziness and confusion. A swinging mirror may cause vertigo and it cannot be too often repeated with emphasis that vertigo is a disturbance or partial loss of consciousness.

As a practical test, the condition of having a dead labyrinth, the author believes, may be rather an advantage than a disadvantage so far as flying is concerned, because normal labyrinths merely afford the possibility of vertigo and dizziness. Some authorities, such as Von Cyon, even contend that the inner ear can give no information as to the body's relation to the vertical or to space.

The author further calls attention to the fact that in our instinctive concepts of balance, the lateral balance, from side to side, is the usual one, and that it rarely fails. The forward and backward equilibrium is much less secure, and when we fall it is usually in one of these two directions. Thus confusion or dizziness is usually attributed to false horizontal motion. The sense of the vertical, it would seem, is called on to a greater degree in aviation. Possibly also the vertical semicircular canal plays a more important rôle. It would be instructive to learn, the author thinks, whether there is such a thing as rotational vertigo in a vertical, as well as horizontal, plane.

Normal distinct vision, as determined by the test card with the formuls 20/20, is a natural requirement. Other qualifications are also important : hypersensitiveness to bright light; sharpness of the sense of motion; the acuity of vision in lowered illumination; the appreciation of contrast in form, color, and light; the rapid and accurate judgment of distance, direction, size, and — depending upon all these — space.

